# Structural and compositional evolution of FePt nanocubes in oganometallic synthesis

**DOI:** 10.1186/1556-276X-9-615

**Published:** 2014-11-14

**Authors:** Changwang Zhang, Hanbin Wang, Yuping Mu, Jun Zhang, Hao Wang

**Affiliations:** 1 Hubei Collaborative Innovation Center for Advanced Organic Chemical Materials, Faculty of Physics and Electronic Science, Hubei University, Wuhan 430062, People’s Republic of China

**Keywords:** FePt nanocubes, Oganometallic synthesis, Composition, Heterostructures, Growth behavior

## Abstract

In this study, the mechanisms for the formation of FePt nanocubes via pyrolysis of iron pentacarbonate [Fe(CO)_5_] and platinum(II) acetylacetonate [Pt(acac)_2_] were investigated. The time evolution of the structure, morphology, and composition of the FePt nanocubes was probed by transmission electron microscopy (TEM) at different reaction stages. On the basis of the detailed characterization, we determined the following aspects of the reaction mechanism: (1) The FePt nanocubes are rapidly formed at 160°C to 180°C by the decomposition of the precursors, and the formation of the FePt nanocubes is dominated by the nucleation of Pt-rich species followed by a slow deposition process of Fe atoms. (2) A thin Fe atomic layer is present on the FePt nanocubes, which does not influence their phase transition into a fct structure. (3) The use of Fe(CO)_5_ is the key factor leading to the anisotropic growth of the FePt nanocubes, and the Fe(CO)_5_/Pt(acac)_2_ molar ratio not only determines the composition of the resulting FePt nanocubes but also affects their morphology and structures.

## Background

There has been enormous research interest in monodisperse FePt nanoparticles in recent years, driven by new applications such as ultra high-density magnetic storage media, biological imaging, therapy, and catalyst [1-5]. Previous reports have demonstrated that spherical FePt nanoparticles can be obtained by simultaneous reduction of platinum(II) acetylacetonate [Pt(acac)_2_] and iron(II) acetylacetonate [Fe(acac)_2_] or thermal decomposition of iron pentacarbonate [Fe(CO)_5_] [6,7]. As the expanded studies, shape-anisotropic FePt nanocrystals such as cubic [8], wirelike [9], and rodlike [10] shapes (nanocubes, nanowires, and nanorods) were fabricated based on the ‘surfactant-assisted mechanism’ in oganometallic method. It is believed that the shape-anisotropic nanocrystals can be applied for new magnetic devices. For example, the FePt nanocubes can be used to achieve special texture and magnetic alignment, which are essential to future high-density magnetic recording [11]. Rather, in the area of catalysis, the catalytic properties are sensitive to the special facets of the nanocrystals [12,13], making FePt nanoparticles a great potential for future application.

Monodisperse cubical FePt nanoparticles (NPs) were fabricated by Shukla et al. by using dichlorobenzene as the solvent, and a net [100] texture of the self-assembly was observed [14]. Meanwhile, Chen et al. reported the synthesis of FePt nanocubes by using benzyl ether as the solvent [11]. They found that the self-assembled arrays had a cubic packing. The shape control of the FePt NPs was also studied by Liu and Chen [15,16], in which the surfactants were found to play a crucial role in controlling the shape of the nanoparticles. Though the shape-anisotropic FePt nanoparticles were reported by a few literatures, the detailed growth behaviors involving the formation of the nanoparticles are not fully understood. For example, it is not clear at which reaction stage the nanostructures determine their shape and how their composition changes during the synthesis. Therefore, it is highly desirable to understand the formation mechanism which is important for the future application of shape-anisotropic FePt nanomaterials.

In the literature, we focus on the structure and formation mechanism of FePt nanocubes prepared using the oganometallic method. The FePt nanocubes were synthesized via simultaneous reduction of platinum acetylacetonate and thermo-decomposition of iron carbonyl in benzyl ether using oleylamine and oleic acid as surfactants. To trace the time evolution of the morphology and composition of the FePt particles, the samples were extracted during different reaction stages and observed by transmission electron microscopy (TEM) after chemical washing. After systematically examining the FePt nanocubes at different reaction stages, we observed that the cubic shape of the FePt NPs was formed in the first 15 min as the reaction begun. Further reaction did not affect the size of the nanocubes, while the composition of Fe in FePt nanocubes increased as the reaction continued. A core-shell structure of the FePt nanocubes is demonstrated in this study. It is also found that the use of Fe(CO)_5_ is a key factor leading to the anisotropic growth of the FePt nanocubes, and the Fe(CO)_5_/Pt(acac)_2_ molar ratio not only determines the composition of FePt nanocubes but also has impact on their structures.

## Methods

### FePt nanocube synthesis

The FePt nanocubes were synthesized by modifying the method reported by Chen [11]. In a typical procedure, 0.5 mmol Pt(acac)_2_, 4 mmol oleic acid, 4 mmol oleylamine, and 2 mmol Fe(CO)_5_ in benzyl ether were heated to 200°C for 5 h under nitrogen protection. The heating rate was kept at approximately 5°C per minute below 200°C. Before the temperature reached 200°C, 1-mL samples were extracted from solutions at 160°C, 180°C, and 200°C, respectively. After the reaction was finished, the five extracted samples and the final product were washed using the same procedures as the following: firstly, an appropriate amount of ethanol was added into the solutions. The products with black color were then precipitated by gentle centrifugation. The yellow-brownish supernatants were discarded. The precipitates were then re-dispersed in hexane and precipitated again in ethanol by centrifugation (3,000 rpm for 5 min). Further purification of the products was performed by dispersing the products into hexane, precipitating with ethanol, and centrifuging. Finally, the purified nanoparticles were dispersed in hexane.

### Material characterization

X-ray diffraction (XRD) was used to determine the crystalline structure of the annealed FePt nanocube powder. The detailed morphology and microstructure of various samples are characterized using TEM (Tecnai 20 ST, FEG, FEI Co., Hillsboro, OR, USA). The compositions of the FePt particles were determined using the energy-dispersive X-ray (EDX) spectrometer attached to the same microscope. Magnetic measurements were performed on the FePt nanocubes using a Quantum Design SQUID magnetometer (Quantum Design Inc., San Diego, CA, USA).

## Results and discussion

**Figure 1:**
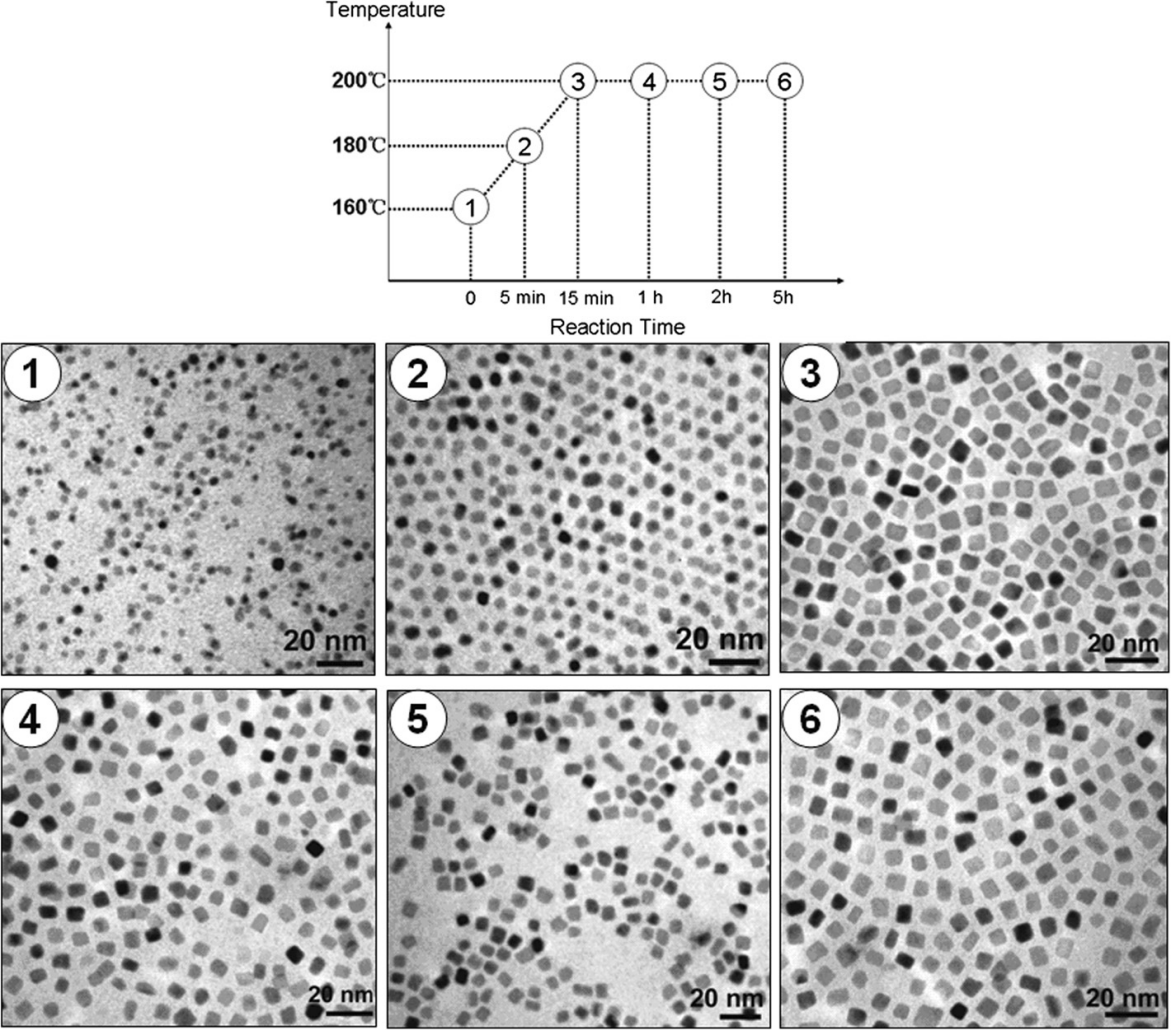
TEM images of the FePt nanocubes at different reaction stages.

**Figure 2:**
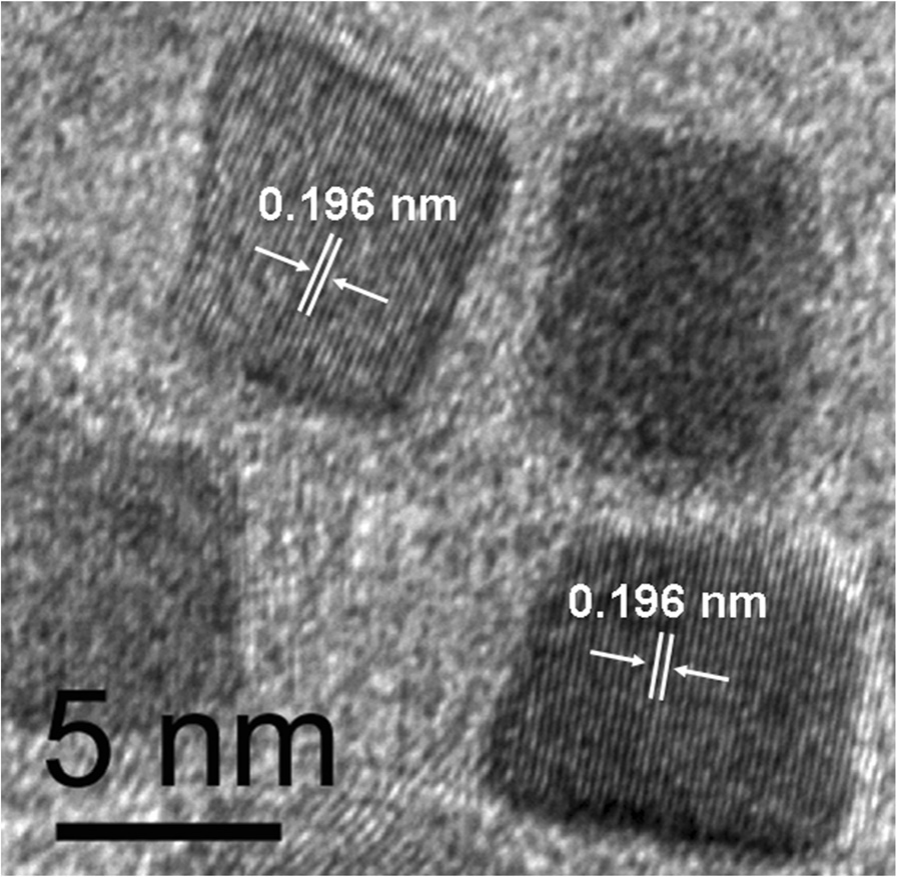
**High-resolution TEM image of the Fe**_**44**_**Pt**_**56 **_**nanocubes.**

Figure 1 shows the TEM images of the FePt nanocubes at different reaction stages. Judging from image 1, the size of particles which reach 3 nm reveals that the reaction has passed the fast nucleation process at 160°C. In the experiments, we had tried to collect FePt NPs at a temperature lower than 160°C; however, no sample was obtained after centrifugation probably due to the ultra-small size of the NPs. The FePt NPs in image 1 show spherical morphology and do not manifest anisotropic growth at this stage. In image 2, as the temperature increased to 180°C, the size of the NPs grew to 4 nm with narrower size distribution. Furthermore, the particles had manifested some degree of anisotropic growth, in which a part of NPs was nearly cubic in shape with corners rounded up. In stage 3, as seen in image 3, the shape of the particles has changed to cubic with side size about 6 nm. After stage 3, as seen in images 4 to 6 in Figure 1, the FePt nanoparticles showed no obvious variation in shape and particle size by extending reaction time, revealing that these characters were determined once the reaction reached 200°C. It seemed that the deposition of Pt and Fe precursors on the nanocubes was negligible after stage 3. The Ostwald ripening process, which usually happens in the growth of nanoparticles, did not occur in the reaction. Shown in Figure 2 is the high-resolution transmission electron microscopy (HRTEM) image of the FePt nanocubes; the inter-fringe distance of the lattice fringes is measured to be 0.196 nm, being close to the lattice spacing of the (200) facet of the face-centered cubic (fcc) FePt alloy. These point to the fact that the {100} lattice fringes are parallel to the edges of the FePt nanocubes, which is consistent with Chen's reports [11].

**Figure 3:**
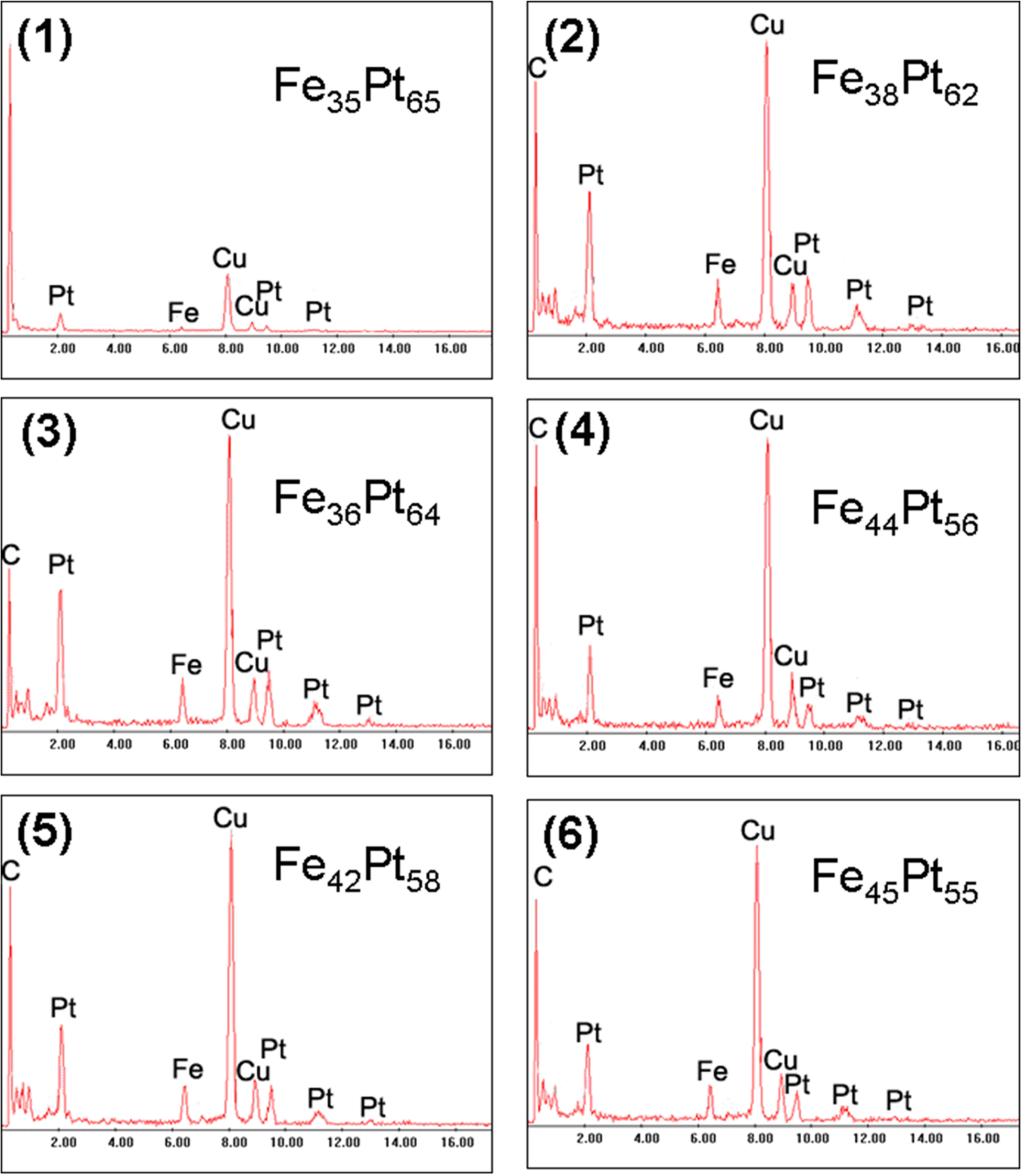
**EDX spectra and calculated composition of the nanocubes in the corresponding six stages in Figure**1**.**

The variation in composition of the nanocubes in the six formation stages was measured by EDX. Figure 3 shows the EDX spectra and the calculated composition of the nanocubes in the corresponding six stages. The composition of the nanoparticles in stage 1 was Fe_35_Pt_65_, indicating that the number of Pt atoms was evidently higher than that of the Fe atoms. The result suggested that the reduction of Pt salt was faster than the decomposition of Fe(CO)_5_ in solutions, resulting in more Pt atoms entering into the embryo in the nucleation process [15]. In stage 2, the composition of the FePt NPs was determined to be Fe_38_Pt_62_, in which the content of Fe atoms was slightly increased due to the accelerated decomposition of the Fe salt. The composition in stage 3 showed no obvious change relative to stage 2, though apparent particle growth was evidenced in stage 3. After the reaction had been proceeded for 1 h, the composition of the FePt particles changed obviously. The composition of the particles was Fe_44_Pt_56_, and no further variations were observed in stages 5 and 6, indicating that the Fe and Pt atoms were consumed in the last two stages.

Compared with the TEM and EDX results in stages 3 to 4, the shape and size of the particles showed no obvious change while the content of Fe atoms in the particles increased. The phenomena suggested that after the formation of FePt nanocubes, a thin Fe layer was likely deposited onto the nanocubes due to subsequent decomposition of Fe precursors. The thin Fe layer has only several atomic layers, and it has little influence on the size of the particle but will increase the content of Fe in FePt nanocubes due to the size effect. For example, if we suppose that the FePt nanoparticles are cubic, we can calculate an average shell thickness by comparing the composition in stages 3 and 4. The value we obtain, 0.2 nm, corresponds to one Fe layer. The FePt/Fe core-shell structure was reported by a few literatures [17,18]. However, these reports were only limited to spherical nanoparticles. The report on the core-shell-type FePt nanocubes might show opportunities in their potential applications.

With regard to the formation of the fcc-structured FePt nanoparticles, the cubic shape could be attributed to the anisotropic growth of the FePt seeds. If the growth rate of the {111} plane is faster than that of the {100} plane, the {111} plane would disappear due to overgrowth and the {100} plane would be exposed, which determines the cubic shape of the FePt nanoparticles [16]. Similarly, if the growth rate of the {111} and {100} planes is approximately equal, spherical nanoparticles would be formed due to isotropic growth of the different crystal planes. Our observation is in agreement with the kinetically controlled growth mechanism. However, it is not clear whether the Fe atoms at the surface diffuse into the FePt lattice to form a homogenous alloy at relatively low temperature. We have tried to detect the iron layer by HRTEM; however, due to the lattice match between FePt and Fe nanocrystals and because the Fe atom layer is less than 0.5 nm, it is difficult to observe the Fe shell directly. In order to prove the existence of the Fe shell, we used a solvothermal method to strip the Fe atoms based on the dissolving-crystallization process of crystals. In detail, after centrifugation and washing, the synthesized FePt nanocubes were dispersed in 18 mL hexane and put into a 20-mL autoclave. The autoclave was heated to 180°C for 24 h. At this high temperature, the Fe atom would dissolve into the hexane due to the dissolving-crystallization process and consequently cause the decrease of the Fe content in global FePt nanocubes. Additional file 1: Figure S1 gives the TEM images and EDX spectra of the FePt nanocubes before and after solvothermal treatment. It is seen that little is changed in the morphology and size of the particles. However, the composition of the Fe_45_Pt_55_ dropped to Fe_36_Pt_64_ after systematic measurements. It means that the surface of the FePt nanocubes is very likely made of the Fe atoms.

**Figure 4:**
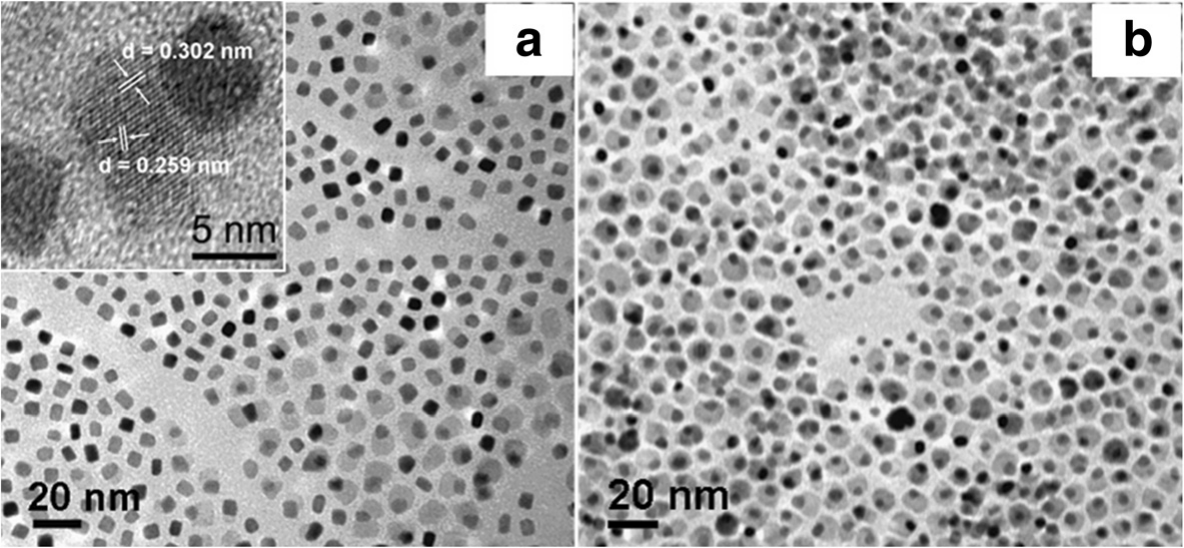
**The synthesized FePt/Fe**_**3**_**O**_**4 **_**heterostructures under the conditions (a) Fe(CO)**_**5**_**/Pt(acac)**_**2**_ **= 5:1 and (b) Fe(CO)**_**5**_**/Pt(acac)**_**2**_ **= 8:1.** The inset in **(a)** shows the HRTEM image of one FePt/ Fe_3_O_4_ heterodimer.

The effects of the reaction parameters such as surfactants and temperature on the size and shape of the nanoparticles have been explored by several research groups [8,9,16] and need not be discussed here. Here, we emphasize that the use of Fe(CO)_5_ is a key factor leading to the anisotropic growth of FePt nanostructures. In our complementary experiments, the use of Fe(acac)_2_ or FeCl_2_ failed to prepare FePt nanocubes. It is inferred that the peculiar chemical reactivity of the Fe-oleic acid complex in a hot organic solution enables the subsequent deposition of Fe on the Pt-rich cores [19]. In the kinetic growth process, the Fe atom preferred to deposit on high-surface-energy facets which eventually dictated the geometric features of the FePt nanoparticles. It is interesting to note that the excess of Fe(CO)_5_ in the reaction does not introduce a thicker Fe shell on FePt nanocubes but yields FePt/Fe_3_O_4_ heterostructures. Shown in Figure 4 are the synthesized FePt nanostructures under the condition Fe(CO)_5_/Pt(acac)_2_ = 5:1; the FePt/Fe_3_O_4_ heterostructures are observed among FePt nanocubes. Further improving the Fe(CO)_5_/Pt(acac)_2_ to 8:1 led to more FePt/Fe_3_O_4_ heterostructures in the products. In the reaction, the existence of Fe(II) or Fe(III) ions was attributed to the oxidation of Fe(0) species by reduction of the Pt(acac)_2_ precursor to Pt(0) [17] and by reacting with oxygen in air [20,21]. An HRTEM image of one FePt/Fe_3_O_4_ heterodimer is shown in Figure 4. The measured interplanar distances for the brighter particle are very close to 0.302 and 0.259 nm, which match well with the ideal values of the (220) and (311) planes for the inverse spinel cubic Fe_3_O_4_. These observations indicated that the activation energy for the generation of Fe_3_O_4_ nuclei in benzyl ether was much higher than the kinetic barrier for enlargements of pre-existing FePt seeds. Hence, it led to the heterogeneous growth of Fe_3_O_4_ on FePt.

**Figure 5:**
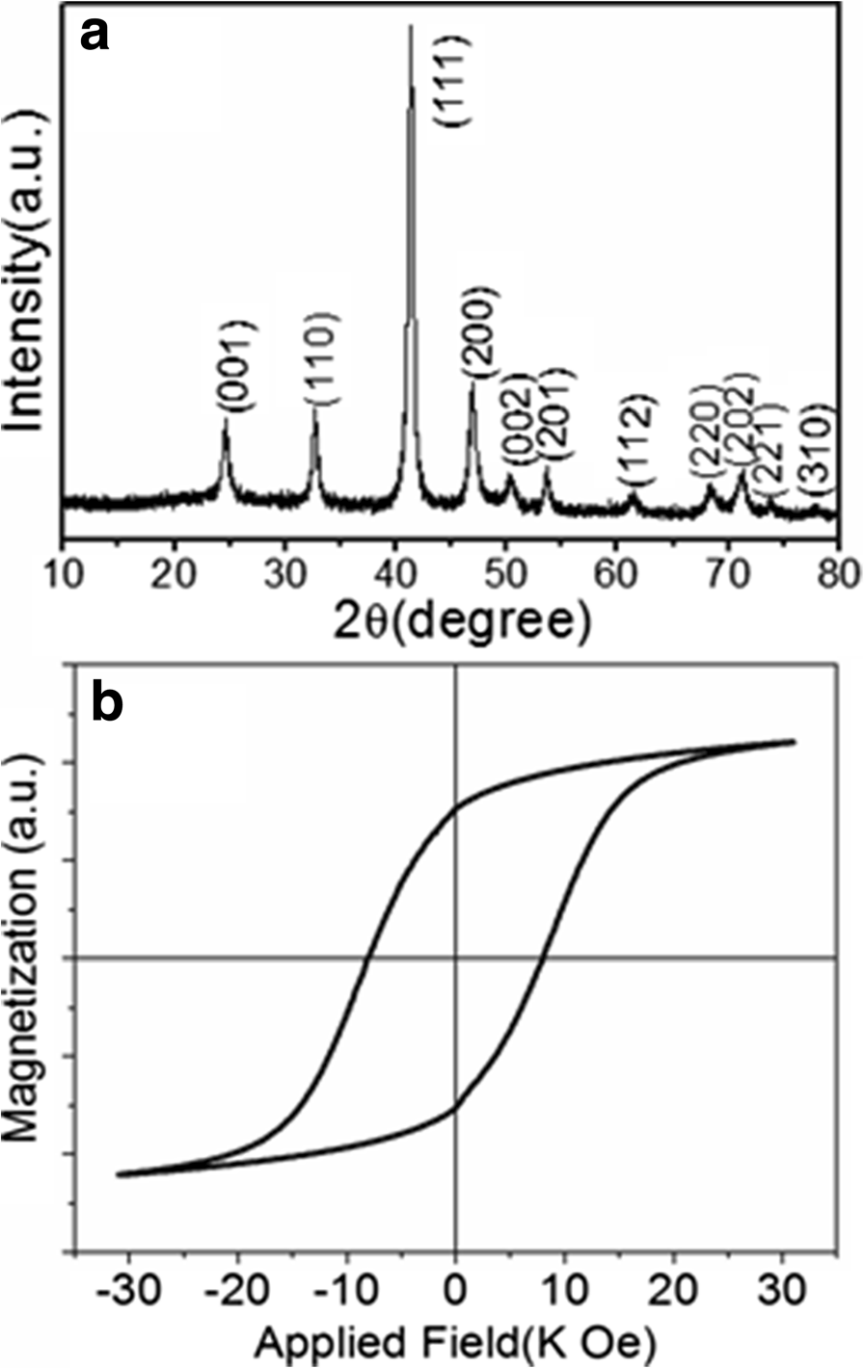
**XRD pattern and room-temperature hysteresis loop. (a)** XRD pattern and **(b)** room-temperature hysteresis loop of the 6-nm Fe_44_Pt_56_ nanocubes after annealing at 650°C for 1 h.

The as-synthesized Fe_44_Pt_56_ nanocubes were annealed at 650°C for 1 h in vacuum in the experiments. The XRD pattern of the annealed sample is shown in Figure 5a; the clear (001) and (110) peaks as well as the shift of (111) peak from 40° to 41° indicate that ordered L1_0_ structure is formed. Moreover, the narrow peaks in the XRD spectrum suggest the growth of FePt particles during annealing. Figure 5b shows the hysteresis loop of the sample in Figure 5a. The coercivity of the particles is 8.2 kOe at room temperature, which is consistent with the XRD results. It is suggested that the FePt core-Fe shell structures do not influence the phase transition of the fcc nanocubes, which is consistent with the previous report on spherical FePt NPs [17]. Under high temperature annealing, the Fe atoms at FePt cubes diffused into the nanocube to form a homogenous nanoalloy, and the global composition at 40% < *x* <60% would ensure the fcc Fe_*x*_Pt_1 - *x*_ nanocubes changing to a fct structure.

## Conclusions

To clarify the growth behavior for the formation of FePt nanocubes synthesized by the pyrolysis of Fe(CO)_5_ and Pt(acac)_2_, we systematically investigated the variability in the morphology, composition, and structure of the nanocubes at different reaction stages. We evidenced by TEM and EDX analysis that the nanocubes had a core-shell structure consisting of a Pt-rich core and one Fe monoatomic shell. The use of Fe(CO)_5_ was a key factor leading to the anisotropic growth of FePt nanostructures through subsequent deposition of Fe atoms on the high-surface-energy (111) facet. The excess of Fe(CO)_5_ did not increase the thickness of the Fe shell on FePt nanocubes but led to the heterogeneous growth of Fe_3_O_4_ on the FePt nanocubes, wherein the process was governed by the kinetic growth mechanism.

## Competing interests

The authors declare that they have no competing interests.

## Authors’ contributions

HBW and CZ performed the experiments, analyzed the data, and drafted the manuscript. The whole project was under the direction of HaoW, who designed the experiments and revised the manuscript. JZ and YM participated in the synthesis and characterization of the FePt nanoparticles. All authors read and approved the final manuscript.

## Supplementary Material

Additional file 1: Figure S1 (a, b) TEM images of the FePt nanocubes before and after solvothermal treatment at 180°C for 24 h. (c, d) The corresponding EDX spectra of samples (a) and (b). Click here for file
