# Supplementary figures and images for: Structural and compositional evolution of FePt nanocubes in oganometallic synthesis
Source: Nanoscale Res Lett. 2014 Nov 14;9(1):615. doi: 10.1186/1556-276X-9-615 (PMC4236211; doi:10.1186/1556-276X-9-615)

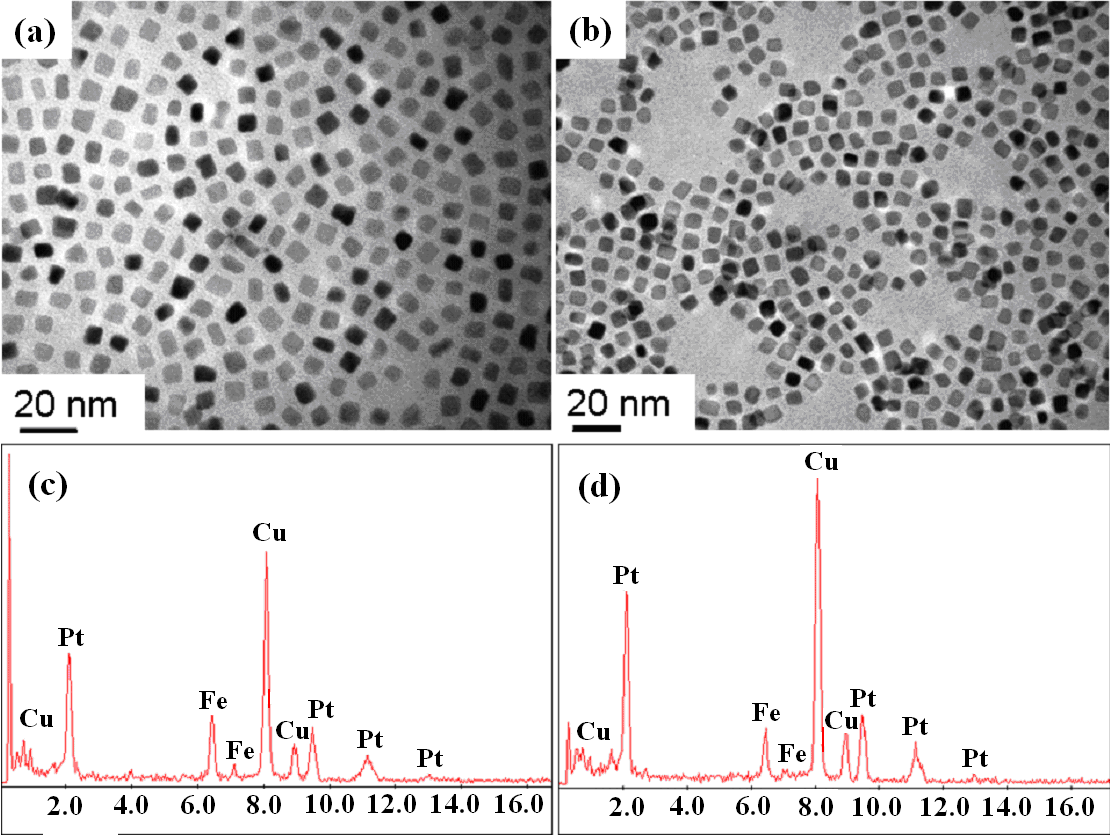

Supplement: Additional file 1: Figure S1 — (a, b) TEM images of the FePt nanocubes before and after solvothermal treatment at 180°C for 24 h. (c, d) The corresponding EDX spectra of samples (a) and (b). [file 1556-276X-9-615-S1.tiff]
